# The Royal Society: Present and Future

**DOI:** 10.1155/S1463924692000166

**Published:** 1992

**Authors:** Michael Aityah


Journal of Automatic Chemistry, Vol. 14, No. 3 (May-June 1992), pp. 69-72

### The Royal Society: Present and Future

*The following, which we feel will be of great interest to our readers both in academia and industry, is the text of the Anniversary Address to the Royal Society, given by the President, **Sir Michael Aityah**, on 29 November 1991.**

When I took over as President this time last year I realized that I had a very difficult act to follow. Under Lord Porter the Royal Society had expanded its role in various directions and was playing a prominent part in national affairs. As one aspect of this I found myself immediately involved in setting up and steering a major Science Inquiry. While I might have preferred a gradual introduction into all the complex issues involved, I have instead undergone what can only be described as a crash course. The Science Inquiry, which has a very broad remit, has solicited views from a wide range of individuals and organizations. As a result the Society has first-hand knowledge of the views of the scientific community and can clearly identify the issues that cause most concern.

Digesting this material and producing a measured report will take a few more months. Some of the issues on which the Society is focusing include the position of the individual scientist at all stages in the scientific process. There are many serious questions about the recruitment, pay and conditions of Ph.D. students. This leads on to the even more difficult problems concerning postdoctoral fellows and others on short-term appointments. The size of this group, as compared with tenured staff, has increased dramatically over the past decade, and this raises fundamental issues which must now be addressed.

Manpower planning, as an attempt to forecast and influence the future, has had a bad name, but it would be irresponsible not to examine retrospectively the manpower consequences of lack of planning. There are thousands of young scientists who have been attracted into the beginnings of a research career who have no clear idea what the future holds for them. They need to be advised and assisted so that they can make intelligent and realistic plans for their careers.

The role of the individual scientist is also fundamental when we come to the organization and funding of science. Many scientists feel that, for various reasons, the role of the individual has diminished as more emphasis is put on projects and programmes. Perhaps we need to look for more mechanisms which will strengthen the hand of the individual scientist.

The Royal Society has, of course, been doing its share in this direction through its research appointments: Research Professorships and University Research Fellowships (URFs). At present there are about 180 of the latter in post and the scheme has been remarkably successful in identifying talent at a crucial stage, since the bulk of URFs succeed in getting permanent appointments at UK universities well before the expiry of their full fellowship period. The whole operation works very smoothly thanks to the great efforts put into it by our Biological Secretary, Professor Brian Follett.

Perhaps the greatest tribute to the URF scheme is that it has the enthusiastic support of absolutely everyone, all the way from the grass roots to the ABRC. The only complaint is: why don't we have more of them?

As a mathematician, I am conscious of my somewhat singular position in being the President of the Royal Society. A number of my illustrious predecessors were mathematicians, but the practice seemed to die out about 100 years ago with Sir George Stokes. I take my presence here today to be a reaffirmation of the basic role mathematics plays in the sciences, although this role is a constantly changing one. In addition to its traditional and intimate relation to all the physical sciences – the A-side in our jargon – it is increasingly finding application on the B-side. The recent award of the prestigious Balzan Prize to Professor John Maynard Smith reminds us that mathematical models have taught us something about the process of natural selection. The spread of epidemics lends itself to sophisticated mathematical analysis. Problems of vision and visual interpretation involve several areas of science, but there is certainly an important mathematical component. In all these fields old mathematical ideas are finding new applications and new branches of mathematics are being created to attack new scientific problems.

We all know that the traditional barriers between scientific disciplines have been rapidly breaking down, and this tends to emphasize the unifying role of mathematics. Perhaps one of these days the Royal Society will even abolish its division into an A-side and a B-side!

But mathematics is not restricted to the natural sciences. It plays an increasingly important part in the social sciences, particularly in economics. The younger generation of economists tends to be skilled in mathematics, and a young mathematician who covets a Nobel Prize could do worse than move into the field of economics. In a different direction, mathematics has a traditional link with logic and philosophy, a link which has acquired greater importance through the growth of computer science.

All of this shows clearly that mathematics stretches well beyond the present boundaries of the Royal Society and into fields covered by the British Academy. For this reason I particularly welcome the friendly co-operation

* Previously published as a supplement to *Royal Society News*, Volume 6, Number 6, November 1991, and reprinted with the permission of The Royal Society.

© 1992 Sir Michael Aityah that exists between the Royal Society and the British Academy. In other countries the word 'science' is interpreted in a much broader sense, and in dealing with our colleagues overseas I have found the narrower remit of the Royal Society to be an embarrassing constraint. Partnership with the British Academy is the obvious way of dealing with this problem.

It is not only in international affairs that the Royal Society should make common cause with those who work outside the natural sciences. Most of us, on both sides of the divide, work in universities and, as we all know, universities in this country are under severe pressure. The past decade has seen financial stringency coupled with greater Government direction, and there is every indication that the next decade will produce even greater challenges for universities. At this critical juncture it is, I believe, essential for all academics to act together and not take a sectarian view of their own narrow interests.

I would therefore like to use this occasion, of my first Anniversary Address to the Society, to examine some of the crucial questions that face the universities in the next few years. My audience in this respect is not just those Fellows of the Society and their friends who are present in this room, but all our colleagues in the universities and, even more, those involved in the decisions which will affect our future.

Over the past decade or so universities have increasingly been treated like commercial businesses, dominated by accountancy procedures and measured by the products they produce. Elaborate formulas have been produced, labelled with symbols such as DR, SR and JR, which have become compulsory jargon for all Vice-Chancellors. The President of the British Academy put it well when he said recently that the great divide in the universities now is not between the Arts and the Sciences, but between the scholars and the accountants.

Now there will be those who will say that this is the reaction of unrealistic academics, who do not realize that universities have to be properly managed to the satisfaction of the tax-payer. Perhaps – but I, and I suspect many university colleagues, are deeply uneasy about the present situation, not because we are irresponsible, but because we have little confidence in the foundations on which the great accountancy edifice has been built. As a mathematician I know that the validity of a conclusion rests not only on the accuracy of the argument but also on truth of the initial premise. In all the intricate discussions that take place on university funding I feel I want to question the initial starting point, not the arcane details of the mechanisms.

So, where do we start? We are told that universities have two functions: to teach (T) and to conduct research (R). These activities are supposed to be more or less independent and their products measured by counting student degrees or published papers. With this simple, not to say simplistic, starting point one then proceeds to work out detailed figures for different institutions depending where they sit in the RT plane. It is beautifully simple as an algebraic accounting procedure, but does it correspond to reality?

All of us have had to fill in forms asking us to identify how much of our time, in a given period, has been spent on teaching and how much on research. For many of us this is a meaningless and impossible task, especially when one moves from elementary to more advanced teaching. How does one divide, count or weigh a thought? In desperation we end up by filling in some notional figures that give the expected kind of answer. These then are the murky beginnings of the grand edifice that is ultimately constructed, when figures, percentages and graphs are produced to provide a spurious accuracy for the whole process.

I am sure I am not alone in my scepticism. Universities are complex intricate structures that cannot adequately be described or assessed by two algebraic variables.

If I had to use a two-word definition, I would prefer to say that universities are places of *Learning* and *Thinking*. Everyone in a university, from the most junior student to the most senior professor, is engaged in both these activities. Learning covers the whole spectrum, from the acquisition of vocational skills to the higher scholarship at the frontiers of knowledge. The common pursuit of learning is the spirit that animates the university and gives it its integrity. Across the centuries and across the disciplines this has been the central function of universities.

If learning is the objective, thinking is the process. Critical and creative thought are the life-blood of universities, the indispensable route to the acquisition of true knowledge, and the essential ingredient in the education of our citizens.

Of course I am not seriously proposing one simplistic formula to replace another simplistic formula. My point is that there are many dimensions to a university and we must be aware of the consequences of over-simplification.

Let me return now to the standard terminology of Research and Teaching, the framework in which all current debate takes place. No one disputes that Research is a vital part of our university activities, but it is as well to recognize that, while the objective of all Research is the acquisition of new knowledge, the procedure varies enormously from discipline to discipline. It is hardly necessary to spell out the difference in character between research in science on the one hand and research in the humanities on the other. Moreover, within these broad categories there are major differences from subject to subject, between experimentalists and theorists, between mathematicians and biologists, or between economists and classicists.

I think it is necessary to emphasize these differences because each of us develops views which are inevitably rooted in our own experience. This is as it should be, since second-hand views do not carry the same conviction or authority. The danger, however, is that we are tempted to extrapolate from our own experience to fields far from our own, to assume that a research policy or structure that is suitable for physics applies equally well to chemistry, mathematics or ancient history. As a mathematician I

will have my own perspective and my own limitations, which I acknowledge in advance. But since mathematics occupies a basic role in theoretical science, and since it straddles the divide between the arts and the sciences, the perspective of a mathematician may be usefully different from that of an experimental scientist. Naturally I will be biased in favour of people and brains, as against large laboratories and expensive equipment, but perhaps the time has come to re-examine the balance between man and machine.

Let me try to summarise what I see as the fundamental problem for research in universities; let me ask: how have we reached our present position and what are the dangers ahead?

In the past, research or the pursuit of learning was conducted in universities as an integral part of their normal activities, and did not require large sources of external funds. Newton was supported by Trinity College and the University of Cambridge, not by any early version of the SERC. However, in the past 50 years or so, research in experimental science has become increasingly expensive, with high-energy physics setting a trend which has, in varying degrees, spread to most scientific disciplines. This has led to our present system of research councils, with its increasingly dominant role in the support of university research in the sciences. To oversimplify, the research councils support, through specific grants, research in universities over and above what the universities would normally carry out on their own.

In the early days research council support to universities was a welcome and essential aid for research in certain fields.

By now the scale and scope has shifted to such a degree that the tail is wagging the dog. Research tends to be identified with those activities supported by the research councils, and the idea that research is an integral and normal part of university activities is rapidly being lost.

As a mathematician, whose need for equipment is modest, I am very concerned at the way that the arguments about research and its future funding tend to focus almost exclusively on expensive experimental science. Here I believe I am also speaking for all our colleagues in the humanities and social sciences, those fields which, in the economist's terminology, are labour-intensive rather than capital-intensive. For all us intellectual labourers our main concern is that we should be allowed the freedom and time to think and create. There is a real danger that our needs will be overlooked or swamped by a new bureaucratic wave of reform.

Perhaps the most dangerous threat at present lies in the increasing pressure for selectivity or concentration of resources. For subjects which need expensive equipment, some concentration is inevitable and desirable. The danger is that a general philosophy in favour of concentration in all subjects will come to be accepted, even though it is neither necessary nor appropriate. I fail to see, for example, why mathematical research should not be encouraged in all universities and I am sure that colleagues in the humanities would have similar views.

You might think that all this hardly needs saying or that I am exaggerating the dangers. On the contrary, those of you who have your ears close to the ground in Whitehall will know that major changes are just months away. The impending abolition of the binary line, between polytechnics and universities, has led the UFC to embark on a new selectivity exercise which has profound implications for the universities. There will be strong pressures for a further separation of R and T, and many university departments may end up being financed for teaching only. What this means in particular is that the staff–student ratio in such departments will be set at a level designed to occupy all the time and effort of the staff. The traditional view, that research is part of the purpose and duty of university staff, will be discarded.

There are some who would consider such a change mildly regrettable but not a disaster. They argue that research and teaching are not closely connected and that it should be perfectly possible to run a high-level establishment on a teaching-only basis. I am not so sanguine. One has only to look at the situation in our schools to appreciate that teaching has a hard time competing with more lucrative professions. Universities are still able to recruit high-calibre staff, despite unfavourable salaries, precisely because they offer the intellectual rewards embodied in the pursuit of learning. But to put the argument on a loftier plane, I feel that an institution whose staff no longer have aspirations to make their own contributions to higher learning, has lost any claim to be considered a university.

This is not to say that teaching is an unimportant part of a university's activities nor that all staff have to be engaged throughout their careers on fundamental research. On the contrary, I do not see any clear line between teaching and research and I prefer to view them both as part of the process of learning. I see this as an open-ended process which leads, at various levels and through various channels, from the known to the unknown. Everyone entering a university should feel that there are no limits to intellectual inquiry and exploration. Nothing could be so inhibiting and discouraging as to be told that your teachers are not part of this great intellectual adventure.

I am aware of the complex relations between schools, universities and employers, and of the need for universities constantly to adapt themselves to changing circumstances. There is certainly scope for universities to examine their traditional methods of instruction and to question old habits.

What is needed is a flexible varied system that provides for the needs and aspirations of a much larger proportion of our population than is currently catered for. The vision of a university that I have been describing is at one end of a continuous spectrum of institutions that must have diverse roles and aims.

Many of the changes in education that are being mooted are welcome. Some are less so. In particular I view with some alarm suggestions that there should be two-year degree courses. The arguments in favour of such courses are seductive: normal academic terms are quite short, so a more intensive course using up slack vacation time should be able to achieve as much in two years as a normal course does in three. The fallacy in this argument is best seen by trying to apply the same reasoning to school education. Perhaps we could turn out the same product at age 12 instead of 16! The point is that a university education is not just a question of mastering facts and passing tests. Students mature intellectually at university, as they do physically at school, and time is needed for this process. No other country considers that even three years is adequate and we must, in educational as in economic matters, consider how far our practices harmonize with those of our neighbours. A two-year degree would very much be a step in the wrong direction, though two-year courses with less ambitious aims would be perfectly reasonable.

All the questions I have touched upon, and many others, will be looked at in detail by a new Royal Society working group on higher education under Sir Eric Ash.

### International affairs

The Society has extensive links with academies and other scientific bodies all round the world, and Sir Anthony Epstein is a tireless traveller on the Society's behalf. He has also been extremely active in setting up new exchange agreements with other countries and, what is more difficult, persuading appropriate bodies to provide the necessary funds. He retired in November 1991 having served five years. He will continue to be active in international scientific circles through his involvement with ICSU and other bodies.

His successor as Foreign Secretary is Dr Anne McLaren who has already been active in Society affairs in a number of capacities.

The past few years have seen momentous changes in Eastern and Central Europe, and the Society has been actively following up the opportunities which have arisen. We now have fellowship programmes with Hungary, Czechoslovakia and Poland to bring young postdoctoral fellows to this country for a period of one year. These are funded by a combination of Government and private funds, and allow for up to 12 fellowships annually for each of the three countries. The fellows apply individually and are selected by a joint panel which includes our Foreign Secretary. The standards of the applicants are encouragingly high.

The Society is also helping some of these countries to reorganize their scientific structures. Delegations have visited the UK, under our auspices, to see at firsthand how our scientific arrangements work. These have been much appreciated and we hope to extend this kind of assistance to other countries.

Changes in Eastern Europe are more recent and even more dramatic. Many uncertainties remain, particularly with regard to the status of the Soviet Academy of Sciences, but the fragmentation of the Union has already led to the independence of the Baltic States and to parallel movement by the other Republics. As a result the scientific academies in many of the Republics have approached the Royal Society, and we have had visits by delegations from the three Baltic States, Georgia and the Ukraine. The Society's aim is to continue, and if possible to increase, the scientific exchanges with all parts of the former USSR. Developments are monitored as closely as possible with the aim of offering any assistance that may be needed to help Soviet science and scientists over the difficult years ahead.

International matters of a different sort arise in our Group on Scientific Aspects of International Security, which is jointly chaired by Sir William Hawthorne and Professor Martin Rees. The turbulence of international affairs has made the work of this committee even more pertinent and it is currently concerning itself with questions relating to the control of chemical and biological weapons. The Royal Society also hosted a three-day international meeting at Trinity College, Cambridge, devoted to this whole area.

Also held at Trinity College this September was the biennial meeting between the Officers of the Society and the Officers of the US National Academy of Sciences. These meetings provide a valuable forum for an exchange of views and information, and provide a possible platform for joint action. One issue on which joint action is planned is that of population. We intend to issue a joint statement, arising from the Cambridge meeting, drawing attention once again to the problems posed by the continuing growth of world population. This is an issue which has a strong bearing on environmental and ecological problems which will be the focus of the international meeting in Rio de Janeiro next year. The Royal Society and the US Academy also hope to arrange, in conjunction with other interested parties, a major international conference on population some time in 1993.

The current initiatives of the Society in connexion with population problems have been led by our Physical Secretary, Sir Francis Graham-Smith, and we are grateful to him for adding this to his many other responsibilities on behalf of the Society.

The meeting with our American counterparts gave the opportunity of learning at first hand about the activities of the US National Research Council (NRC). Sponsored jointly by the National Academy of Sciences, the National Academy of Engineering and the Institute of Medicine, the NRC is a large organization which produces detailed and extensive reports on a wide variety of issues in science and technology. These reports are usually commissioned by some branch of the US Government by Congress. We were impressed by this activity of the Academy and are considering whether the Royal Society should attempt to follow the US lead. Some of our present activities may be seen as modest steps in this direction.

